# Asymmetric two-dimensional ferroelectric transistor with anti-ambipolar transport characteristics

**DOI:** 10.1186/s11671-023-03860-2

**Published:** 2023-06-06

**Authors:** Yilin Zhao, Mengshuang Chi, Jitao Liu, Junyi Zhai

**Affiliations:** 1 CAS Center for Excellence in Nanoscience, Beijing Key Laboratory of Micro-Nano Energy and Sensor, Beijing Institute of Nanoenergy and Nanosystems, Chinese Academy of Sciences, Beijing, 101400 China; 2 School of Nanoscience and Technology, University of Chinese Academy of Sciences, Beijing, 100049 China

**Keywords:** Ferroelectric transistor, Van der Waals heterostructure, Anti-ambipolar, Negative transconductance, Coupling effect

## Abstract

Two-dimensional (2D) ferroelectric transistors hold unique properties and positions, especially talking about low-power memories, in-memory computing, and multifunctional logic devices. To achieve better functions, appropriate design of new device structures and material combinations is necessary. We present an asymmetric 2D heterostructure integrating MoTe_2_, h-BN, and CuInP_2_S_6_ as a ferroelectric transistor, which exhibits an unusual property of anti-ambipolar transport characteristic under both positive and negative drain biases. Our results demonstrate that the anti-ambipolar behavior can be modulated by external electric field, achieving a peak-to-valley ratio up to 10^3^. We also provide a comprehensive explanation for the occurrence and modulation of the anti-ambipolar peak based on a model describing linked lateral-and-vertical charge behaviors. Our findings provide insights for designing and constructing anti-ambipolar transistors and other 2D devices with significant potential for future applications.

**Supplementary Information:**

The online version contains supplementary material available at 10.1186/s11671-023-03860-2.

## Introduction

Significant attention has been given to two-dimensional (2D) ferroelectric transistors that rely on 2D ferroelectric materials in the development of low-power data storage [1, 2], neuromorphic computing [3, 4], and multifunctional logic devices [5, 6]. Ferroelectric materials possess unique qualities such as remnant polarization, which make them useful in applications including data storage, sensors, and micro-electro-mechanic systems (MEMS). Yet, due to size limitations and surface defects [7], traditional ferroelectric materials pose difficulties in achieving high dimensional controlling ability and stability. 2D van der Waals ferroelectric materials such as SnS [8], In_2_Se_3_ [9], CuInP_2_S_6_ (CIPS) [10], however, exhibit excellent surface flatness and enhanced controlling ability, providing a more dependable foundation for device optimization [11]. The high surface atomic ratio of 2D ferroelectric materials can regulate the interfacial effects which have the potential to improve heterojunction properties, making 2D ferroelectric transistors a promising device structure.

One of the electrical behaviors of special interest is the anti-ambipolar characteristic. It has been widely studied and adopted in frequency multipliers [12, 13] and multi-value logic devices [14, 15], among other applications in circuit managements [16, 17]. Anti-ambipolar transistors are distinguished by their Λ-shaped (inverted V-shape) of transfer curves and the presence of both positive and negative transconductance. The p-n heterojunction transistor structure was previously used to build anti-ambipolar transistors, wherein a p-type semiconductor is attached in series with an n-type semiconductor following energy band matching, including WSe_2_/SnS_2_ [18], tetracene/MoS_2_ [19], MoTe_2_/MoS_2_ [12, 20], WSe_2_/ReS_2_ [21], to name a few. Nevertheless, the carrier concentration of both p-type and n-type semiconductors is low within the conducting region, which results in low peak currents, and weak electrical modulation effects.

In recent years, Cheng et al. [22] proposed an anti-ambipolar transistor based on an asymmetric van der Waals heterojunction structure that was already established by Li et al. [23] They investigated the operation mechanism and applications of the anti-ambipolarity. The semi-floating gate structure with tunneling effect of channel electrons under electric field was deployed, to attain a high peak current at the μA level, and a strong electrical modulation effect. However, the device did not display strong anti-ambipolar characteristics under negative drain bias.

Herein, an anti-bipolar device utilizing an asymmetric two-dimensional ferroelectric transistor structure is introduced. As a compliment to previous methods, this device has realized anti-ambipolar electrical transport traits under both positive and negative source bias, by exploiting structure asymmetry along with ferroelectric properties. The device exhibited a peak current reaching a level of 100 pA and a peak-to-valley ratio of three orders of magnitude. These results pave the way for more research and development of anti-ambipolar transistors.

## Methods

### Device fabrication

The device is fabricated through dry-transfer technique. Multilayered CIPS flake with a straight edge is mechanical exfoliated onto a polydimethylsilothane (PDMS) film, then transferred on the surface of a piece of heavily p-doped silicon substrate with 300 nm of oxide layer, which is cleaned by sonication in Acetone and deionized (DI) water before. Then, h-BN and MoTe_2_ flakes are transferred over the CIPS flake in sequence using the same method. After each transfer process, 8 h of vacuum annealing at 80 ℃ is conducted to promote the adhesion between each layer. The MoTe_2_ channel can be divided into two parts by the edge of CIPS. Bulk CIPS, h-BN, and MoTe_2_ materials are purchased from SixCarbon Technology, Shenzhen. The source and drain electrodes are then patterned on the sides of MoTe_2_ channel via electron beam lithography (EBL) and fabricated by electron beam evaporation (EBE) deposition of 20 nm Cr and 50 nm Au. The channel length is about 6 μm. All the crystals, flakes and samples are stored in a vacuum dryer.

### Device characterization

The morphology of fabricated devices was obtained by optical microscope (Olympus BX51M). The thickness of the materials and the device was characterized using a atomic force microscopy (AFM) (Asylum Research MFP-3D) system. The scanning electronic microscopy (SEM) image was obtained using a scanning electronic microscope (FEI Nova NanoSEM 450). Piezoelectric force microscopy (PFM) measurements were performed on 7 wt% Nb-doped SrTiO3 substrates using dual AC resonance mode. All electrical properties were investigated by a Keithley Semiconductor Parameter Analyzer (4200) in conjunction with a vacuum probe station (Janis ST-500-UHT-4TX-6PORTS). All tests were conducted at fixed temperature of 300 K in vacuum.

## Results and discussion

**Fig. 1:**
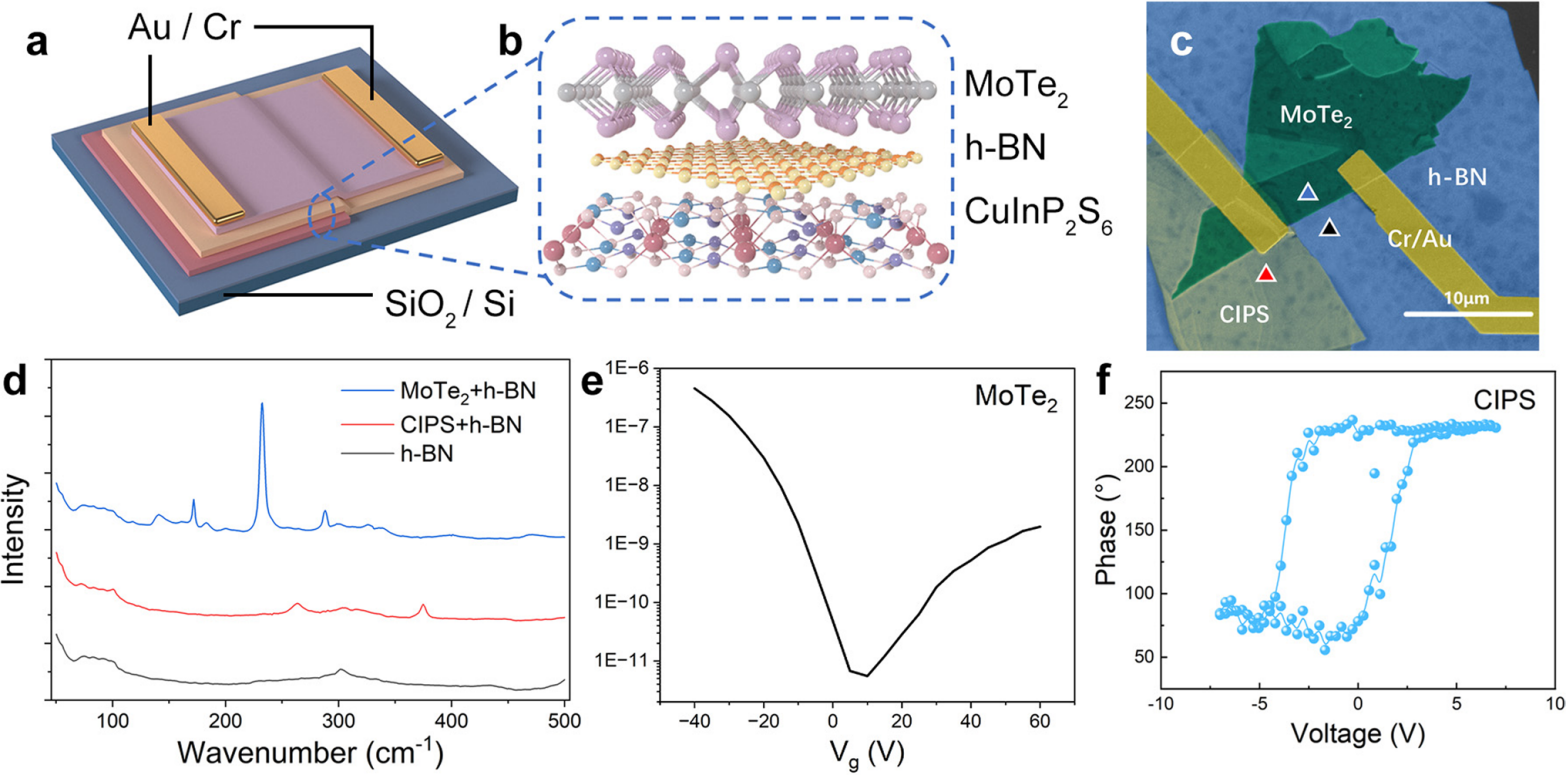
Structural design of the device. 3D schematic of **a** the device and **b** the heterostructure. **c** Pseudo-colored SEM image of the device. The scale bar is 10 μm. **d** Raman spectroscopy results of different spots marked in (**c**). **e** Transfer curve of intrinsic MoTe_2_ transistor. **f** On-state hysteresis of CuInP_2_S_6_ (CIPS) flake by DART-PFM

As the 3D schematic diagram of the device shown in Fig. 1a, an asymmetric van der Waals heterostructure is constructed on silicon substrate, consisting of CIPS, isolating layer h-BN, and MoTe_2_ channel, and the stacking structure is magnified and illustrated in Fig. 1b. It is worth notifying that the channel region is divided into two parts by the edge of CIPS. An electrode is deposited on the part of MoTe_2_ channel above CIPS (MoTe_2_-C), and the other electrode is on the part without CIPS (MoTe_2_-NC).

To further illustrate the actual visual effect, a pseudo-colored SEM image is displayed in Fig. 1c. And Raman spectroscopy results of different spots are shown in Fig. 1d, with the scanned spots marked in Fig. 1c. The test results show that a strong absorption peak near 220 cm^−1^ is visible in the Raman spectra of the blue spot in the MoTe_2_ region, which can be matched with the position of the MoTe_2_ E_1g_ peak; the spectra of the red spot in the CIPS region have a small absorption peak near 260 cm^−1^ and 370 cm^−1^ each, which can be matched with the positions of the S–P–S peak and the P–P peak, respectively [24]. Since both materials are within the range covered by h-BN, the absorption spectra of pure h-BN were also compared and confirmed that there is no significant absorption peak within the detection range. The thickness of each layer in the device is characterized by AFM. The thickness of MoTe_2_ is about 12 nm. A 1.8-nm-thick h-BN is assumed to be around 3 atom layers. As for the CIPS layer, an approximately 50-nm-thick flake is obtained to ensure the ferroelectric effect. The AFM results are displayed in Fig S1 (Supporting Information).

The selection of materials for each layer of the device was done with deliberate consideration. Ambipolar semiconductor MoTe_2_ was chosen for its well-researched properties, which enable the channel to generate both holes and electrons when subjected to an external electric field. The ambipolar field-effect characteristics of the intrinsic MoTe_2_ transistor can be seen in Fig. 1e. The MoTe_2_ channel is partially controlled by the CIPS flake, which functions as a ferroelectric gate. The CIPS flake is located between the silicon substrate and the source electrode, allowing for effective polarization to be achieved when a voltage bias is applied to the silicon bottom gate while the source is grounded. Ferroelectric characterization of a multilayer CIPS flake on a 0.7 wt% Nb-doped SrTiO_3_ substrate was conducted using dual AC resonance mode piezoelectric force microscopy (DART-PFM). Figure 1f shows a counter-clockwise hysteresis, indicating the ferroelectricity of the fabricated CIPS flake. The coercive electric field of CIPS was found to be approximately 2.5 V. For the device electrodes, Cr and Au were the preferred materials, with Cr being commonly used as an adhesion layer owing to its work function of 4.6 eV. Located between the valence and the conductive bands of MoTe_2_, Cr renders MoTe_2_ viable for use as an ambipolar semiconductor when they are in conjunction. Moreover, the contact between the Cr and the semiconductor results in less resistance, which enhances the overall efficiency of the device [25]. Therefore, 20 nm of Cr and 50 nm of Au are used here to ensure the contact.

**Fig. 2:**
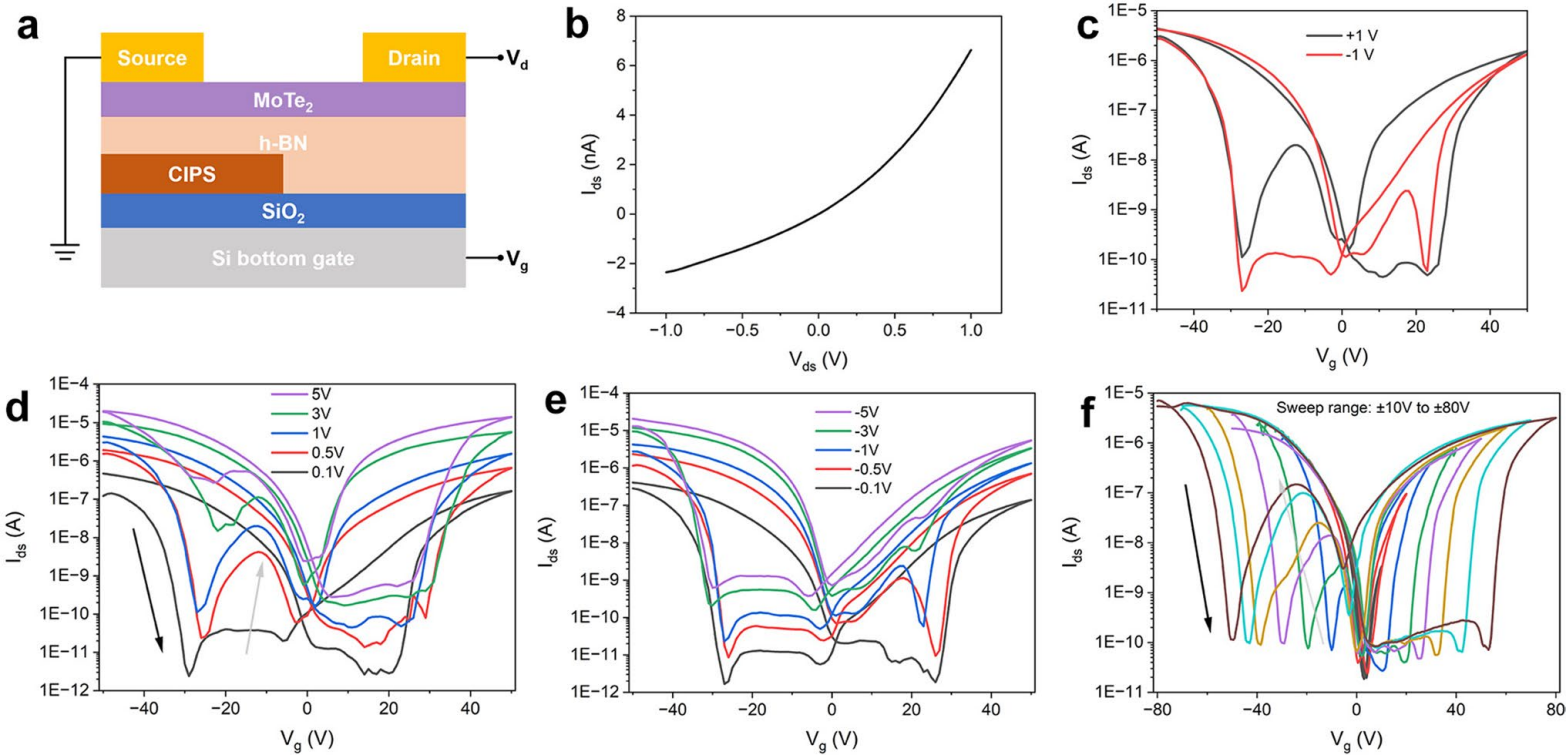
Basic electrical properties of the device. **a** Schematic of the testing system. **b** Output curve of the device without *V*_g_. Dual-sweep transfer curves with** c** ± 1 V of *V*_ds_ and ± 50 V of *V*_g_ sweep range, different **d** positive and **e** negative *V*_ds_, and **f** different *V*_g_ sweep range

The investigation commenced with an analysis of the basic electrical properties. A testing system, as depicted in Fig. 2a, was employed, wherein the source electrode was grounded and positioned above CIPS, while the drain electrode was employed as the other electrode. The ferroelectric layer and the channel were simultaneously impacted by the applied gate voltage (*V*_g_) on the Si substrate. Since the device responds to ambient environments (Fig S2 and Fig S3, Supporting Information), all the tests in the manuscript are carefully conducted in vacuum. Subsequently, the drain-source current (*I*_ds_) curve was analyzed against voltage bias (*V*_ds_) within the range of ± 1 V, and the result is presented in Fig. 2b. The plot style indicated Ohm contact between the channel layer and the electrodes. The device’s gate leakage current (*I*_g_) level was characterized via a dual-sweep scan of the *V*_g_ in the ± 50 V range. The resulting *I*_g_–*V*_g_ curve of the device is illustrated in Fig S4 (Supporting Information), indicating a consistently low *I*_g_ value within ± 50 pA throughout the test.

Continuing the investigation, the transfer characteristic curve was dual-swept with ± 1 V of *V*_ds_ and ± 50 V of *V*_g_ sweep range, as shown in Fig. 2c (with the arrow indicating the sweep direction). The observed curves exhibited two hysteresis windows in the positive and negative *V*_g_ regions for both forward and reverse voltage bias, indicating the presence of charge storage behaviors. This phenomenon signifies the crucial role played by CIPS ferroelectric layer under the present device structure. Additionally, h-BN is also capable of storing charges. However, the hysteresis induced by h-BN differ in the positive and negative *V*_g_ ranges, and the memory window is much narrower [26]. At symmetrical voltages, the negative *V*_g_ side had a slightly higher current level than the positive *V*_g_ side, indicating that the MoTe_2_ utilized in this device demonstrates p-type doping propensity, displaying stronger hole conductivity than electron conductivity in the top of bipolarity. Furthermore, both sweeps revealed a distinctive scanning peak labeled the anti-ambipolar peak due to its resemblance to the classical features.

A series of experiments were conducted to investigate the properties of the anti-ambipolar peaks. Firstly, transfer characteristic curves of the device were tested within the same sweep range but different *V*_ds_, and the results are presented in Fig. 2d, e. The occurrence of hysteresis windows and peak positions of anti-ambipolar peaks remain unchanged and are not affected by *V*_ds_, except when *V*_ds_ is as low as 0.1 V, where no anti-ambipolar peak is observed. The changing mode for positive and negative *V*_ds_ is almost the same, and the peaks are located on opposite sides of the origin. It could be inferred that the migration, tunneling, or recovery of only one type of charge may not induce the peak; therefore, the mechanism here is proved different from former research. However, the absence of the peak in the sweep of the opposite direction suggests that there must be a relationship between the peak and the behavior of carrier charges.

Further analysis was carried out on the transfer characteristic curves at different scan ranges. From Fig. 2f, it is observed that there is no hysteresis window in the transfer curves within ± 20 V of the scan range. This indicates that the ferroelectric layer may not play a charge-constraining role or produce polarization flip during the scanning test within ± 20 V *V*_g_, and the maximum electric field at this time is also consistent with the electric field corresponding to the coercive field of CIPS in the PFM test. The transfer characteristic curves exhibit a similar pattern when scanned in the ± 30 V range and above. The anti-ambipolar peak can be observed in the negative *V*_g_ range of the forward scan, and there is also the presence of a hysteresis window in both the negative and positive *V*_g_ ranges, which grows wider as the sweep range enlarges. The center position of the anti-ambipolar peak also drifts away from zero along with the expansion of the sweep range. Therefore, vertical charge transfer process other than polarization and depolarization of CIPS should be involved here.

**Fig. 3:**
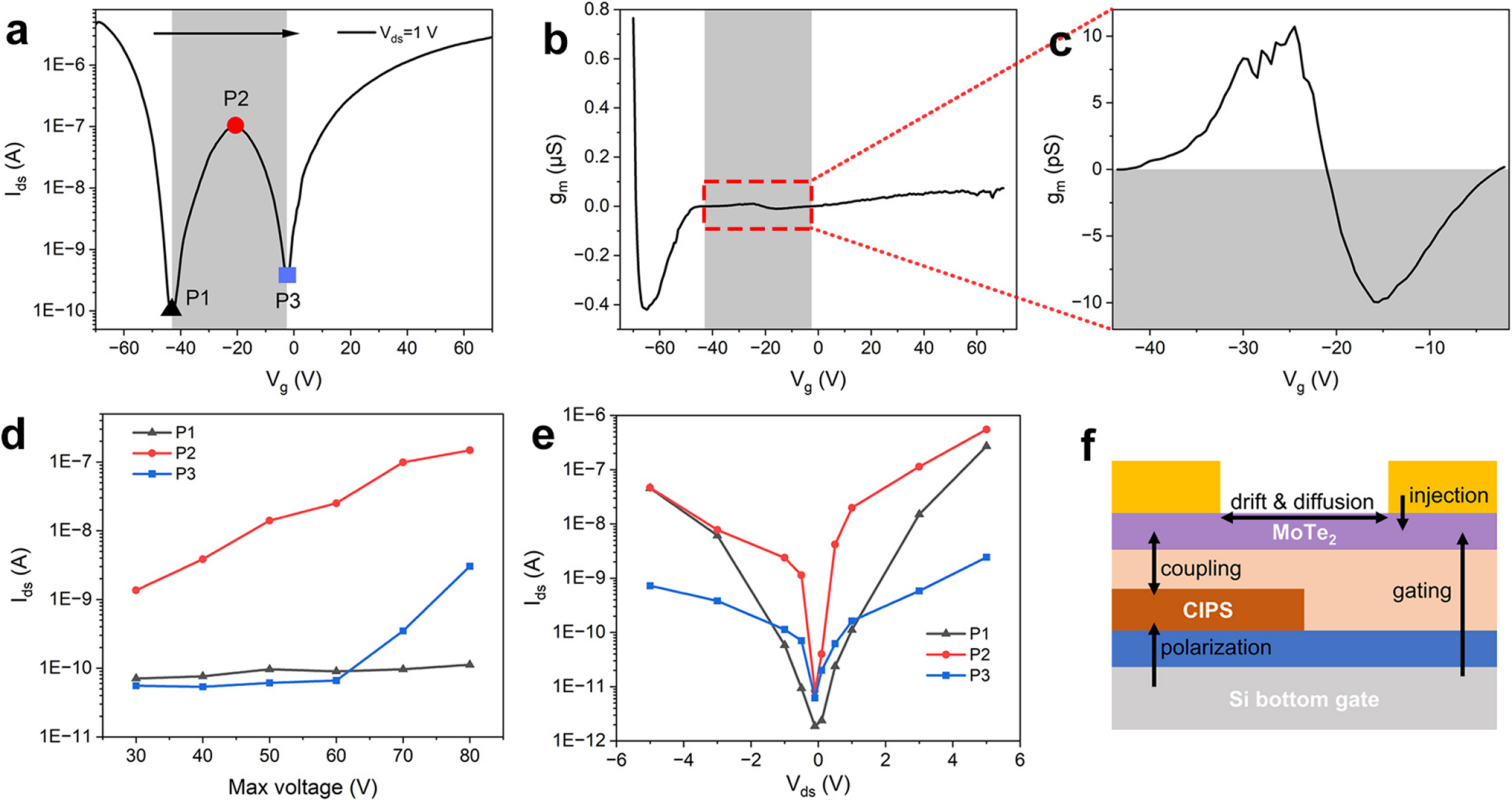
**a** Forward sweep in the transfer curve with 1 V of *V*_ds_ and ± 70 V of *V*_g_ sweep range. **b** *g*_m_-*V*_g_ curve derived from (**a**). **c** Magnified curve of the shaded segment in (**b**). The change in three current states of P1, P2, and P3 for **d** maximum scanning voltage and **e** *V*_ds_

The modulation of the appearance and intensity of the anti-ambipolar peak has been achieved, and the next step is to identify the underlying mechanism driving these changes. A typical curve (Fig. 3a) has been extracted for further investigation, using *V*_ds_ = 1 V and a sweep range of ± 70 V. The shaded region indicates the location of the peak. The three points referring to the summit and both valleys of the peak are labeled as P1, P2, and P3, corresponding to the current states associated with the on-voltage (*V*_g_-on), the peak-voltage (*V*_g_-peak), and the off-voltage (*V*_g_-off). By analyzing the plot data, we have derived the relationship between transconductance and scanning voltage, as shown in Fig. 3b. The plot segment within the range of the anti-ambipolar peak is magnified and exhibited in Fig. 3c, where positive and negative differential transconductance (NDT) regions are clearly visible. The points where the transconductance curve intersects the *V*_g_ axis (*g*_m_ = 0 S) correspond exactly to P1, P2, and P3 marked in Fig. 3a, representing the basic characteristics of the anti-ambipolar peak. Subsequently, we have selected the three corresponding current states in each curve in Fig. 2d, 2e, and 2f and aligned them into six plots, reflecting the changing trends of each spot for the maximum scanning voltage or *V*_ds_. The results are presented in Fig. 3d and 3e, where the plots exhibit distinctly different response styles. P3 is also recognized as the threshold point, and the change of correlated voltage is depicted in Fig S5, supporting information, where we confirm that the position hardly drifted during the tests. To understand the working mechanism, all the charge behaviors are depicted in Fig. 3f and categorized into two types: lateral and vertical. The effect of *V*_g_ is primarily observed in the carrier concentration and vertical behaviors, while *V*_ds_ predominantly influences the lateral behaviors. The transport behaviors of carriers, such as injection, drift (along the electric field), and diffusion (along the concentration gradient), are predominantly lateral. Conversely, ferroelectric polarization, field effect, and the coupling effect of tunneling and electrostatic induction are predominantly vertical.

Charge injection mechanisms mainly consist of field emission (for semiconductors with high mobility) and charge diffusion (for semiconductors with low mobility). These effects are typically described using two distinct theories [27].

The efficiency of channel hot-electron injection is described by Bethe effects, which can be expressed by the Richardson constant equation:

$$J_{{{\text{RE}}}} = {\text{AT}}^{2} {\text{e}}^{{ - \frac{{q\Phi_{B} }}{{k_{B} T}}}} {\text{e}}^{{\frac{qV}{{k_{B} T}}}}$$

where *J*_RE_ is the current density of channel hot electron injection, *A* is the Richardson constant, *T* is the temperature in Kelvin, *φ*_B_ is the height of the Schottky barrier, *k*_B_ is Boltzmann's constant, *q* refers to charge of an electron, and *V* is the electric field strength. This term is affected by the relation between Schottky barrier height and *V*_ds_. And we have:

$$\ln \left( {J_{{{\text{RE}}}} } \right)\sim {\Phi }_{B}$$

$$\ln \left( {J_{{{\text{RE}}}} } \right)\sim V$$

The efficiency of charge diffusion is described by the Schottky diffusion theory:

$$J_{{{\text{SH}}}} = J_{D} \left( {{\text{e}}^{{\frac{qV}{{k_{B} T}}}} - 1} \right)$$

where *J*_SH_ is the current density of charge diffusion, *J*_D_ is the saturated current density, *T* is the temperature in Kelvin,* k*_B_ is Boltzmann’s constant,* q* refers to charge of an electron, and *V* is the electric field strength. This term is basically determined by *J*_D_, and we have:

$$J_{{{\text{SH}}}} \sim J_{D}$$

$$\ln \left( {J_{{{\text{RE}}}} } \right)\sim V$$

And the carrier concentration which affects the saturated current can also be described by the equation:

$$p\left( x \right) = p0*{\text{e}}^{{ - \frac{qV\left( x \right)}{{k_{B} T}}}}$$

where *p*(*x*) is the hole concentration at a distance *x* from the source electrode, *p*0 is the intrinsic hole concentration in the semiconductor, *q* is the charge of a hole, *V*(*x*) is the applied voltage at a distance *x* from the source electrode, *k*_B_ is the Boltzmann constant, and *T* is the temperature in Kelvin. This term is susceptive to both *V*_ds_ and *V*_g_, but mainly *V*_g_.

**Fig. 4:**
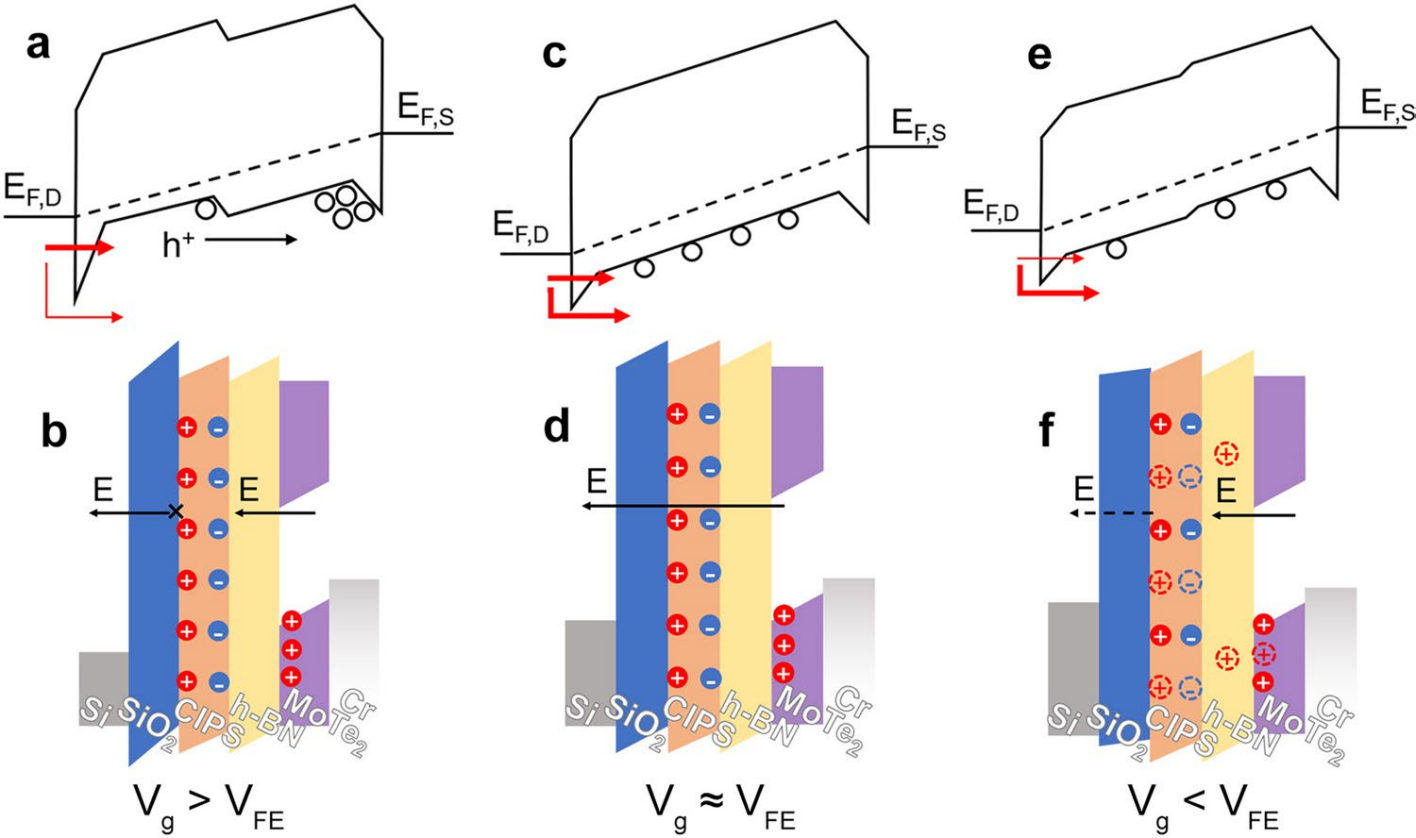
Illustration of lateral and vertical energy band under three stages

Through analysis of the susceptibility of each current state to various directions of modulation, the operation mechanisms can be inferred. In this work, we propose a hypothesis on the mechanism leading to the occurrence of the anti-ambipolar peak. Specifically, we focus on the forward sweep with a positive *V*_ds_, and the energy band of both lateral and vertical under different conditions during the process is illustrated in Fig. 4. It is worth mentioning that all the experiments are conducted under the same temperature in vacuum, fitting the required conditions of thermal equilibrium.

Initially, a negative *V*_g_ with a large magnitude generates a plethora of holes in the MoTe_2_ channel, thereby polarizing the CIPS flake. From Fig. 4a and 4b, it is evident that the carrier concentration of MoTe_2_-NC is initially higher than that of MoTe_2_-C owing to the polarized charges in the ferroelectric layer that may screen the gating electric field. For a p-type channel, a higher carrier concentration results in an elevated Schottky barrier. At this stage, the drift and diffusion directions coincide, causing a significant number of holes to migrate to the MoTe_2_-C side. Since the carrier concentration is high, a huge current is generated, relating to the very top-left segment of the curve in Fig. 3a. Due to the low charge injection efficiency under low *V*_ds_ along with the mass migration of charge carriers, a depletion area is generated in the MoTe_2_-NC region, ultimately causing a severe drop in the current level, reaching the bottom state P1. Thus, the influence of *V*_ds_ on P1 is more significant than *V*_g_. It can be seen from Fig. 3d and 3e that P1 hardly changed with the max scan voltage, which is also the absolute value of the start, but changed the most against *V*_ds_ among the three states.

As *V*_g_ is decreased, a drop in carrier concentration in the channel is observed. This results in a lower Schottky barrier and an increased injection of holes through thermal emission, as illustrated in Fig. 4c. Additionally, the charge generated in both halves of the channel is nearly identical, allowing for drift to take control. Under such conditions, the channel current level is largely affected by *V*_ds_, which causes holes to migrate toward the source, producing a high current output. As a result, the current rises and reaches the summit point P2. From Fig. 3d, it is clear that the change of P2 is roughly linear with *V*_g_. Thus, the P2 state exhibits an exponential relationship with *V*_g_, which corresponds to the equation defining charge concentration. Notably, the change in P2 with respect to *V*_ds_ also matches the results shown in Fig. 3e, where the saturated current density determines the peak value of current.

As *V*_g_ continues decreasing and a lower electric field than the ferroelectric layer is produced, as depicted in Fig. 4e, the vertical effects become dominant. Fewer charges are generated in the MoTe_2_-NC component, whereas the ferroelectric layer undergoes depolarization. The charge generated in MoTe_2_ is contrary to the upper surface of CIPS, thus aiding in maintaining ferroelectricity. Therefore, a faint reverse concentration gradient is produced, and the current rises with higher *V*_ds_. However, the h-BN layer is also capable of storing charges. In the event of *V*_g_ being insufficiently strong, holes in h-BN are compounded with electrons on the surface of the ferroelectric layer, resulting in depolarization and a reduction in channel current, as shown in Fig. 4f. Figure 3e displays how P3 changes at approximately the same rate as P2, indicating that charge concentration remains the principal factor. Nevertheless, Fig. 3d demonstrates that P3 barely alters prior to *V*_g_ reaching 60 V, but changes significantly thereafter. As the tunneling effect is influenced by vertical barriers, this provides an explanation. A series of tests characterizing the output curves utilizing the device as a source-gated transistor is carried out, with the result shown in Fig S6 (Supporting Information). Positive *V*_g_ is applied here, since h-BN presents better ability to store electrons rather than holes. The results prove that high starting voltages produce a strong tunneling effect that causes compounding and depolarization under low *V*_g_.

Hence, the occurrence and intensity of the anti-ambipolar peak is influenced by a series of linked lateral-and-vertical charge behaviors, which is introduced by asymmetric device structure.

## Conclusion

To ensure optimal device performance, it is critical to adopt appropriate designs for device structure and heterojunction construction. In this context, we propose a new ferroelectric transistor employing an asymmetric 2D heterostructure integrating MoTe_2_, h-BN, and CIPS, which exhibits an unusual property of anti-ambipolar transport characteristic under both positive and negative drain biases. Remarkably, we demonstrate that the anti-ambipolar behavior can be modulated by *V*_ds_ and *V*_g_, reaching a peak-to-valley ratio of three orders of magnitude. We further provide a comprehensive explanation for the occurrence and modulation of the anti-ambipolar peak based on a model describing lateral and vertical charge behaviors. Future progress in simulation is expected to give a more comprehensive view. Our findings provide insights that can guide the future design and application of asymmetric 2D devices and anti-ambipolar transistors, which hold significant potential for memory, logic, computing, and other multifunctional devices.

## Supplementary Information

Supplementary file1

## Data Availability

All data generated or analyzed during this study are included in this published article and its supplementary information file.
