# Supplementary material for: Asymmetric two-dimensional ferroelectric transistor with anti-ambipolar transport characteristics
Source: Discov Nano. 2023 Jun 6;18(1):83. doi: 10.1186/s11671-023-03860-2 (PMC10244305; doi:10.1186/s11671-023-03860-2)
Supplement: Supplementary file 1 — Supplementary file1 [file 11671_2023_3860_MOESM1_ESM.pdf]

Asymmetric two-dimensional ferroelectric transistor with anti-ambipolar transport characteristics

Yilin Zhao[1,2], Mengshuang Chi[1,2], Jitao Liu[1,2], Junyi Zhai[1,2*]

[1]CAS Center for Excellence in Nanoscience, Beijing Key Laboratory of Micro-Nano Energy and Sensor, Beijing Institute of Nanoenergy and Nanosystems, Chinese Academy of Sciences, Beijing, 101400, China

[2]School of Nanoscience and Technology, University of Chinese Academy of Sciences, Beijing, 100049, China

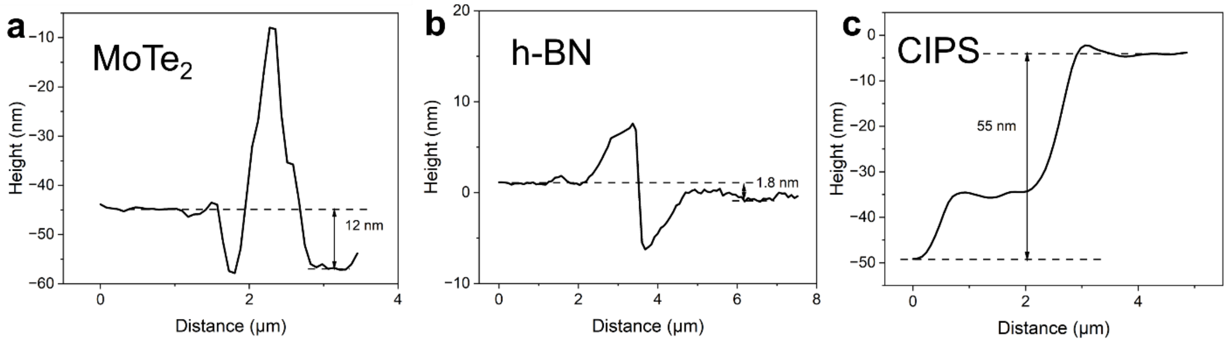

Figure S1. Thickness of **a** $MoTe_2$, **b** h-BN, and **c** $CuInP_2S_6$ (CIPS) in the device.

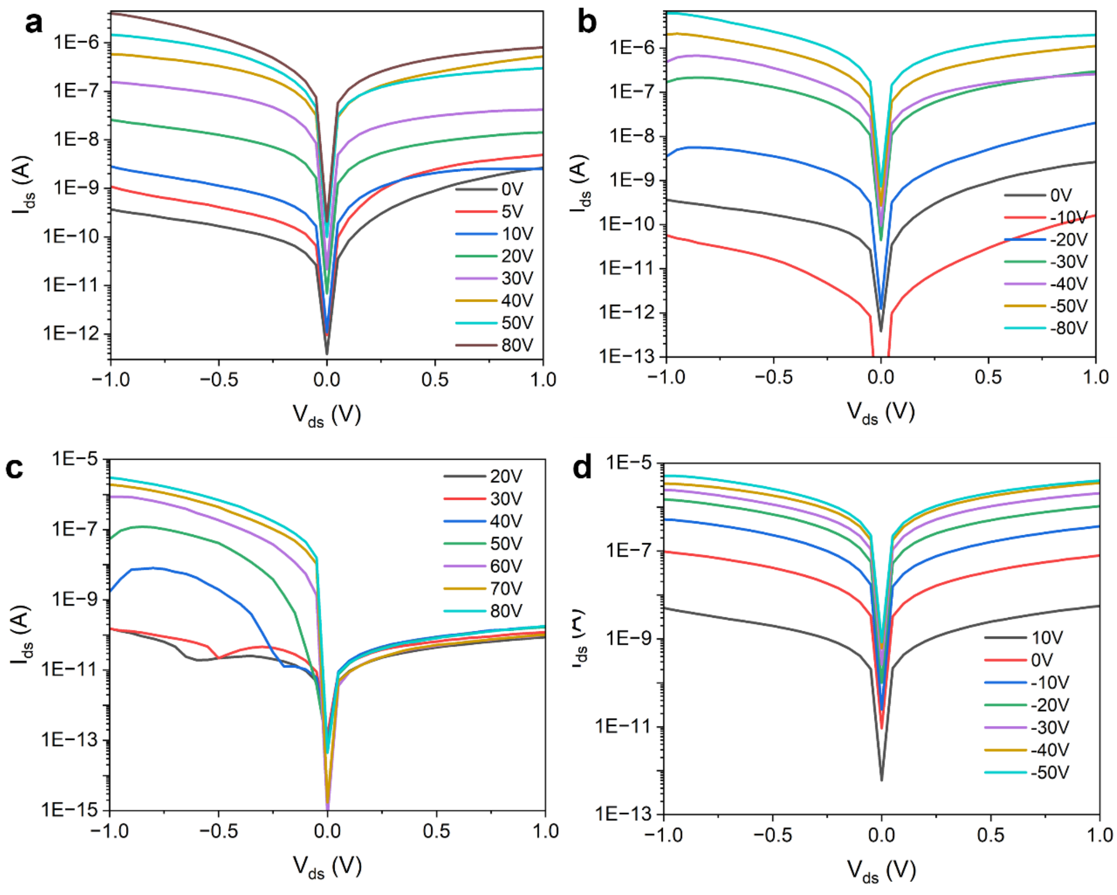

Figure S2. Output curve of the device under different gating voltages in different enviroments. **a-b** Vacuum tests. **c-d** Ambient tests.

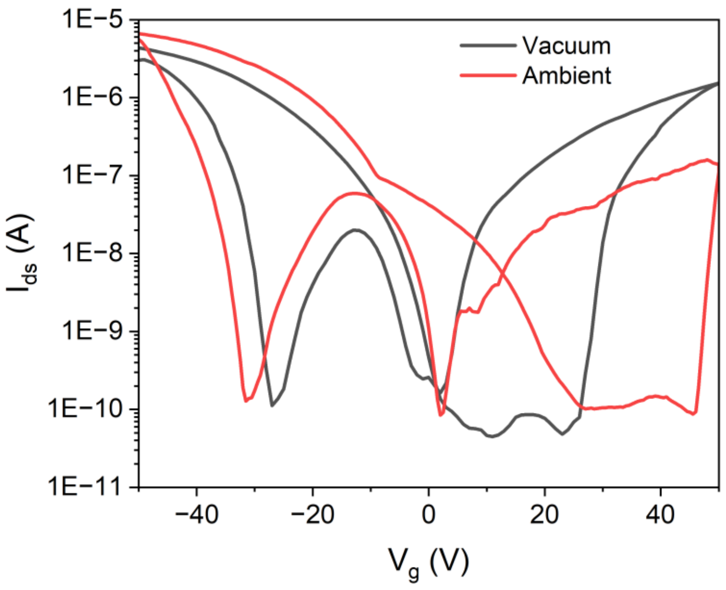

Figure S3. Comparison of transfer characteristics in vacuum and ambient environments. The drain bias is fixed at +1 V and the sweep range is ±50 V.

Note S1. Environmental effects on the device.

As can be inferred from Fig S2, in air, $MoTe_2$ tends to function as p-type semiconductor instead of ambipolar semiconductor in vacuum. Therefore, the environment indeed has great influence on the properties of the device. As can be seen from Fig S3, the transfer characteristics show clear differences between the two environments. In ambient environments, the current is decreased at positive V_g and boosted at negative V_g, indicating the p-type properties induced by Fermi pinning at the electrodes. However, the anti-ambipolar peak remains, and becomes even higher. Therefore, the occurrence of anti-ambipolar peak is not affected by the testing environment.

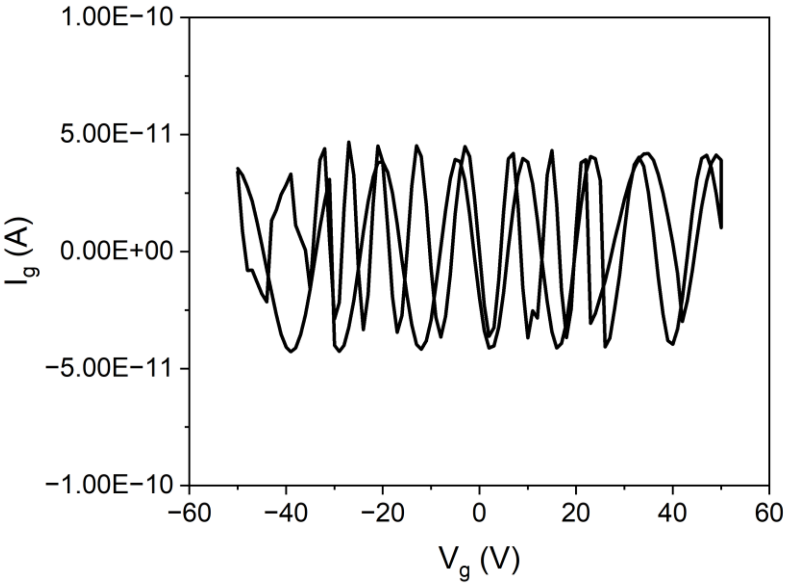

Figure S4. Leaking current curve against V_g

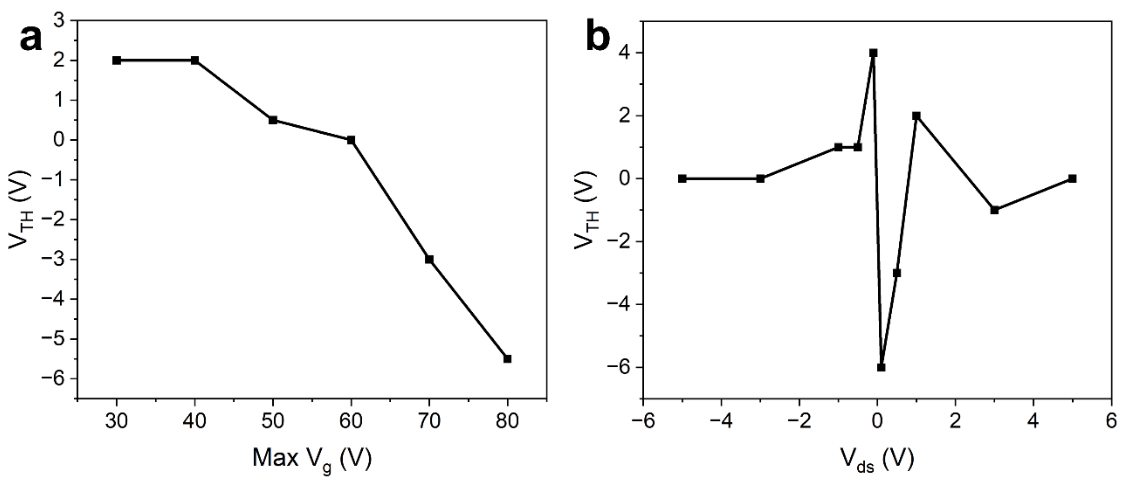

Figure S5. Change in threshold voltage against different **a** maximum V_g and **b** V_{ds}.

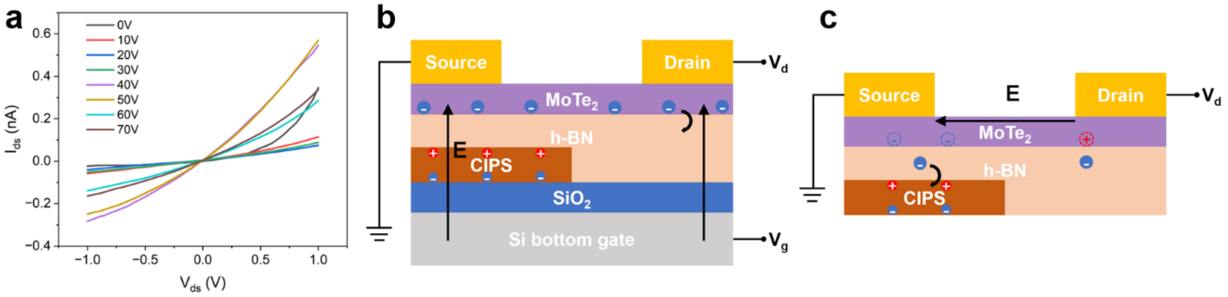

Figure S6. Retention test results of the device. **a** Output curves of the device after applying 10 s of V_g at different strength and withdrawing. **b** Schematic of the charge generation and transfer behaviors in the device with V_g applied. **c** Schematic of the charge behaviors in the device as a source-gated transistor.

Note S2: Retention properties of the device.

Fig S6 presents the results of the retention test, which involves the application and subsequent withdrawal of different values of V_g for a duration of 10 s. Upon withdrawal of V_g, the ferroelectric $CuInP_2S_6$ acts as a gate controlling the barrier on the source side, enabling the device to function as a source-gated transistor. Output curves are presented in Fig S6a, revealing a noticeable rise (from 0 V to 50 V) and fall (above 60 V) in the current level as V_g increases. In the low V_g range, higher values result in stronger polarization retention, whereas depolarization occurs at higher V_g. This change is attributed to the charge-storage properties of h-BN. Under high gating electric field, electrons produced in the channel can migrate to h-BN, as illustrated in Fig S6b. After withdrawal, the remaining electrons combine with the ferroelectric layer and causes depolarization. Additionally, a production of sporadic holes may be induced in the channel. The charge behaviors are described in Fig S6c. As a consequence, lower current levels are observed after high V_g polarization.

Note S3: Charge carrier mobility of the device.

We use the resistance measuring method to calculate the charge carrier mobility in the device. To measure carrier mobility in a semiconductor transistor, the resistance of the device at different applied voltages needs to be measured. The carrier mobility can be calculated using the following formula:

$$\mu = \frac{L}{qnW} \cdot \frac{dI}{dV}$$

where, μ refers to carrier mobility, L refers to channel length of the transistor, q refers to charge of an electron, n refers to charge concentration, W refers to channel width (cm), and dI/dV refers to the slope of the current versus voltage graph.

For newly exfoliated $MoTe_2$ flake, the hole concentration is estimated to be 3.0×10^{11} cm^{-2}.[5] In this work presented in the manuscript, we have L=6.4 μm, W=1.9 μm, dI/dV=2.6×10^{-9} A/V. Therefore, the mobility is calculated to be 0.18 $cm^2/V\cdot s$, which relates to the references.

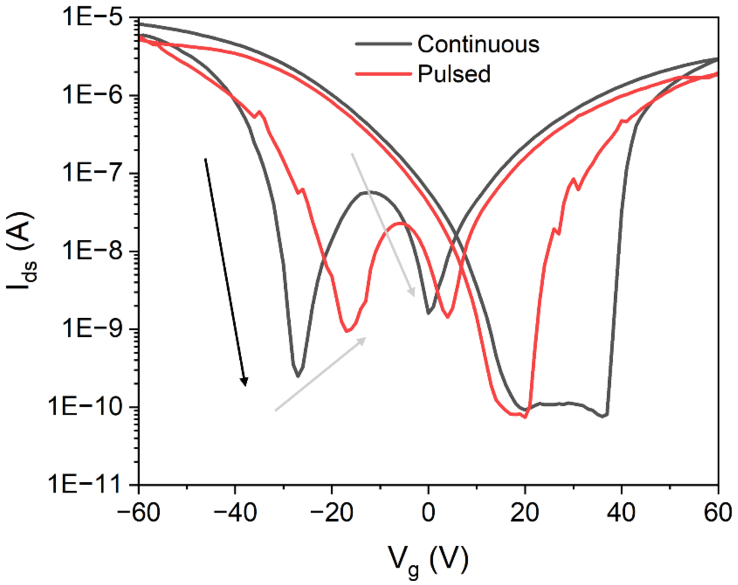

Figure S7. Comparisons of transfer curve obtained with continuous and pulsed V_g.
